# Protocol for determining the contribution of protein and RNA to condensate organization by permeabilizing and enzyme-treating live U-2 OS cells

**DOI:** 10.1016/j.xpro.2025.103744

**Published:** 2025-04-10

**Authors:** Dylan M. Parker, Roy Parker

**Affiliations:** 1 Department of Biochemistry, University of Colorado Boulder, Boulder, CO 80309, USA; 2 Howard Hughes Medical Institute, University of Colorado Boulder, Boulder, CO 80309, USA

**Keywords:** Cell Biology, Cell culture, Cell-based Assays, Microscopy, Molecular Biology, In Situ Hybridization

## Abstract

Ribonucleoprotein granules are comprised of protein and RNA. We present a protocol for querying the relative contribution of protein and RNA interactions to granule organization by permeabilizing and treating cells with proteinase or RNase enzymes. We then detail steps for performing single-molecule fluorescence *in situ* hybridization (smFISH) on enzyme-treated samples for RNA visualization. Finally, we describe detailed instructions for quantifying results generated with this protocol. This protocol can potentially query the contribution of protein-RNA, protein-protein, and RNA-RNA interactions to the organization of any intracellular granule.

For complete details on the use and execution of this protocol, refer to Parker et al.^1^

## Highlights

• A protocol for permeabilizing and enzyme-treating cells in culture
• Steps for performing smFISH and smiFISH on permeabilized samples
• Detailed instructions for quantifying RNA FISH data in degradation experiments

## Abstract (editor highlights)

Publisher’s note: Undertaking any experimental protocol requires adherence to local institutional guidelines for laboratory safety and ethics.

## Graphical abstract

**Figure:**
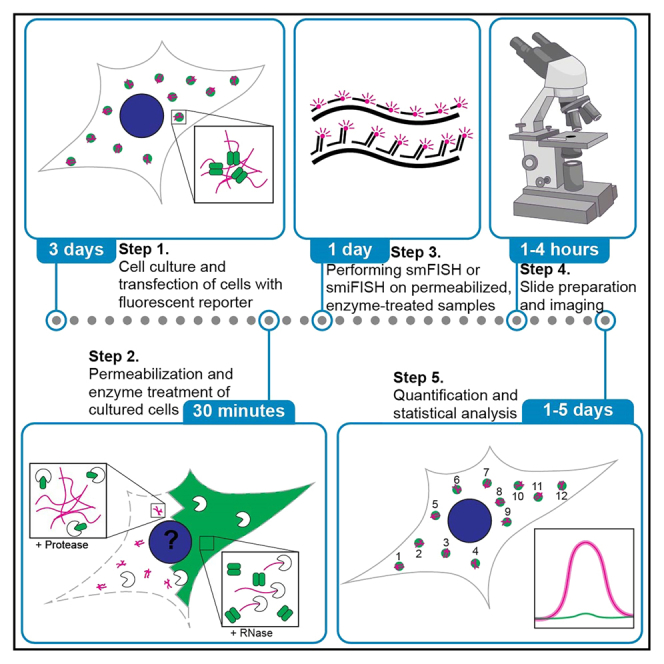

## Before you begin

This protocol provides a workflow for testing the contribution of proteins and RNAs to the ultrastructural organization of RNP granules/membraneless organelles. Specifically, this protocol describes a process for introducing degradative enzymes, such as nucleases and proteases, to living U-2 OS cells using a low concentration Tween-20 permeabilization. Following the degradation of the cellular components of choice, the state of the organelle is examined using fluorescent reporter proteins and single-molecule (sm-) or single-molecule inexpensive Fluorescence *In Situ* Hybridization (smiFISH).^2^^,^^3^ This protocol was recently used to demonstrate that intermolecular RNA-RNA interactions contribute to the organization of stress granules.^1^ Similarly, this protocol was used to show that nucleolar structure is largely retained upon protein degradation.^1^ As this protocol has succeeded in both cytoplasmic and nuclear compartments, we anticipate it will be capable of discerning the structural role of proteins and RNAs in any intracellular membraneless organelle of interest.

Before beginning the experiment, we obtained U-2 OS cells stably expressing G3BP1-GFP protein as a stress granule reporter or a vector for the transient expression of GFP-NPM1 or GFP-Actin to mark nucleoli and the actin cytoskeleton, respectively.^4^^,^^5^^,^^6^ Additionally, we ordered smFISH or smiFISH probes for resident stress granule (*NORAD*, *PolyA*) or nucleolar (*snoRD3A, 47S-ETS1*) RNAs. Investigators should use protein markers and RNA probes relevant to their organelle of interest, as discussed in more detail below. Furthermore, though we have only validated this protocol using U-2 OS cells, it is expected to work with any cell line that is sufficiently adherent to remain attached to coverslips following permeabilization and protease treatment.

### Choosing a protein marker

**Timing: 1 day to ∼2 months**

Choosing the correct marker to examine the role of protein scaffolds in organelle organization is imperative to the success of this protocol. For instance, G3BP1 and G3BP2 are the key organizing proteins that drive stress granule formation.^7^^,^^8^ Loss of signal from G3BP1 during proteinase treatment without loss of SG RNA localization supports a model where G3BP proteins are required for the initial assembly of SGs but not their continued persistence.^1^ When possible, selecting such integral components of an organelle as the protein readout of organization is ideal for investigating their structural role.

Another important consideration is the method of expressing the protein marker of interest. Fluorescent protein knock-in at the endogenous locus is the optimal method for labeling a protein of interest for this protocol. However, it is time-consuming to generate and screen CRISPR lines. Modifying all endogenous loci can also be challenging, considering the abnormal ploidy of U-2 OS cells and other common cell culture lines.^9^^,^^10^ An alternative approach is to create fluorescently labeled protein marker lines using transient transfection, transposition, or viral transduction. Each approach has benefits and detriments, which must be carefully considered and controlled when interpreting results from this protocol. For instance, in the case of transient transfection, transfection efficiency must be very high to ensure individual cells are transfected because, following proteinase treatment, fluorescent markers will be lost. Alternatively, RNA FISH can visualize RNA transcribed from the transfected plasmid as an internal control for cell-specific transfection. Immunofluorescence can be used to visualize unlabeled endogenous proteins; however, it is incredibly challenging to perform combined immunofluorescence/smFISH protocols on permeabilized and enzyme-treated cells without detaching them from coverslips entirely. In short, each method of expressing fluorescent proteins has pros and cons, but typically, knocking in fluorescent tags or stably expressing a fluorescent protein is preferred.

It is also important to consider whether to label the marker protein of interest in wild-type or knockout cell lines. Though overexpressing the fluorescent protein of interest is simple, this can have unintended consequences. Many membraneless organelles are exquisitely sensitive to protein concentration. For example, overexpression of G3BP1 can induce stress granules without exogenous stressors.^11^ Furthermore, the presence of unlabeled protein in proteinase-treated cells can obfuscate their role in organizing cellular compartments. For this reason, using knockout backgrounds is preferable when expressing exogenous fluorescently tagged proteins.

In summary, it is essential to carefully consider the protein marker, expression method, and mode of visualization used in this protocol before proceeding. Deliberate experimental design at this stage will facilitate successful experiments and guarantee robust results. If assemblies are present in unexpected conditions following expression of the fluorescent protein marker, refer to problem 1 in the troubleshooting section.

### Designing and ordering smFISH or smiFISH probes

**Timing: 1 h**

Single-molecule Fluorescence *In Situ* Hybridization (smFISH) functions by hybridizing a series of fluorescently labeled, complementary DNA oligos to an RNA of interest *in situ.* The binding of multiple (typically 48 where feasible) fluorescent oligos to an RNA allows for resolution of single RNAs by microscopy, where RNAs are spatially distinct from one another.^3^

Single-molecule inexpensive FISH (smiFISH) works on a similar principle, with the exception that it uses two DNA oligos: the target oligo has a region that binds to the RNA of interest as well as a second region comprised of a stereotyped “FLAP” sequence.^2^ The fluorescently labeled FLAP oligo is complementary to the FLAP sequence. The benefit of smiFISH is that because the FLAP oligo is complementary to every target oligo, only the FLAP oligo needs to be fluorescently labeled instead of the entire set of RNA-binding primary oligos. The universal secondary design of smiFISH significantly reduces the cost of designing primary probes for new RNA targets, as they can be unmodified DNA oligos with a terminal FLAP sequence.

It is easy to design smFISH and smiFISH probes using one of two free tools: the Stellaris Probe Designer or Oligostan.

1. Identify RNA targeting probe sequences.
  a. Using the Stellaris Probe Designer.
    i. Find the RNA sequence of interest on http://www.ensembl.org/index.html.
      ***Note:*** The sequence of interest should exclude introns when targeting mature RNAs.
    ii. Create an account at https://www.biosearchtech.com/Account/Create.
    iii. Enter the sequence into the Stellaris Probe Designer following the onscreen instructions and click “design.”
    iv. Copy the list of RNA targeting sequences into a spreadsheet.
      ***Note:*** The Stellaris Probe Designer is on version 4.2 at the time of writing this protocol.
  b. Using OligoStan.^2^
    i. Find the RNA sequence of interest.
      ***Note:*** The sequence of interest should exclude introns when targeting mature RNAs.
    ii. Save the RNA sequence of interest as a .fasta file.
    iii. Following the instructions at https://bitbucket.org/muellerflorian/fish_quant/src/master/Oligostan/Documentation/Oligostan_documentation.doc, install the Oligostan R script.
    iv. Replace the file variable in line 3 of the Oligostan script with RNA-of-interest.fasta.
    v. Set the save output location by specifying a destination folder in line 5 of the Oligostan script.
    vi. Run the Oligostan script.
      ***Note:*** Filtered RNA targeting sequences will be saved in the Probes_RNA-of-interest_Filt.txt file in the user-specified output folder.
      ***Note:*** The default Probe Designer and Oligostan settings typically work well. However, if not enough transcript-specific probes can be designed for any reason, changing the masking settings or the number of probes may be necessary. As few as 12 probes can provide adequate signal-to-noise for individual RNA visualization.^2^
2. Finalize RNA targeting probe sequences.
  a. Finalize standard smFISH probes.
    i. If Step 1a is used to identify target probes with the Stellaris probe designer, continue to step 3; the probe designer output sequences will be complete.
    ii. If Step 1b is used to identify target probes with Oligostan, use the RNA targeting sequences in the “Seq” column for smFISH probe ordering.
  b. Finalize smiFISH primary probes.
    i. If Step 1a is used to identify target probes with the Stellaris probe designer, append the desired FLAP sequence to the 3′ end of each probe designer output sequence.
    ii. If Step 1b is used to identify target probes with Oligostan, select the probe sequences from the “HybFlp#” column with the desired FLAP sequence.

***Note:*** As demonstrated in Tsanov et al.,^2^ crosstalk is unobservable between annealed smiFISH probes containing the same FLAP sequence but labeled with different fluorophores. Thus, even if primary probes targeting different RNAs have identical 3′ FLAP Y sequences, they can be annealed in separate reactions to FLAP Y secondary probes labeled with different fluorophores to allow for RNA multiplexing in smiFISH experiments. i.e., if a *snoRD3A* probe set is annealed with an Alexa Fluor 594 FLAP Y secondary probe and *ETS1* is annealed with an Alexa Fluor 647 FLAP Y secondary probe, the RNAs can be distinguished *in situ.*

***Optional:*** Validate RNA targeting sequences by aligning them to the spliced transcript of interest in vector-building software such as Snapgene or A plasmid Editor (ApE).

***Note:*** Though it is typically not necessary to visualize the binding site of targeting sequences, both probe designers design targeting sequences from the 5′ to 3′ direction which can lead to a 5′ labeling bias in longer transcripts. Ensuring probes are homogenously distributed throughout the target can prevent labeling artifacts, particularly in experiments treating cells with directional enzymes or if there are multiple splice isoforms.

***Note:*** When aligning smiFISH targeting sequences, do not include the FLAP region. It does not bind the target RNA.

3. Order FISH probes.
  a. Order standard smFISH probes from Stellaris with a variety of fluorophores by clicking “order” after using the Probe Designer tool or as custom-labeled oligos from IDT.
  b. Order smiFISH primary probes as standard primers from IDT in a pre-diluted 96-well format (see Tsanov et al.^2^ for more details) or as a premixed “oPools” oligo pool.
  c. smiFISH secondary probes can be ordered as custom-labeled oligos from Stellaris or IDT.

***Note:*** The smallest synthesizable quantity of oligos for standard smFISH, smiFISH primary probes, and smiFISH secondary probes is typically sufficient for dozens to hundreds of RNA FISH experiments.

***Note:*** 5′ and 3′ dual fluorescent labeling of smiFISH secondary probes increases the fluorescence roughly linearly but can also increase the background signal if any probes are nonspecific.

***Note:*** The sequences of all smFISH and smiFISH probes used in this study are presented in Table S1.

4. Dilute FISH probes.
  a. Dilute smFISH probes to 12.5 μM in TE buffer.
  b. If received dry, dilute individual smiFISH primary probes to 100 μM in TE buffer.
  c. Combine equal volumes of each smiFISH primary probe into a master mix and dilute to 5X volume in TE buffer (i.e., 96 μL TE to 24 μL primary probe, final volume 120 μL, if using 1 μL each of 24 probes).
    ***Note:*** Regardless of probe number, the final total concentration of primary probes should be 20 μM.
  d. Dilute smiFISH secondary probes to 50 μM in TE buffer.
  e. All types of FISH probes should be vortexed for ∼10–30 s after dilution to ensure they have dissolved completely.
  f. Aliquot and store all FISH probes in the dark at −20°C.

### Annealing smiFISH probes

**Timing: 30 min**

smiFISH takes advantage of universal secondary probes that can anneal to a shared sequence on RNA-binding primary probes to reduce the cost of performing RNA FISH experiments. Because smiFISH probes are bipartite, they require an additional annealing step before being hybridized to RNA *in situ.* This section describes the process of annealing smiFISH probes, which can then be stored at −20°C for at least several weeks before being used in a hybridization reaction.

5. In a PCR tube, prepare the annealing reaction.

**Table:** smiFISH Annealing Reaction Mixture

Reagent	Amount	Final concentration
Primary probe mixture (20 μM)	2 μL	4 μM
Secondary probe (50 μM)	1 μL	5 μM
10X NEBuffer 3 (or 3.1)	1 μL	1X
Water	6 μL

6. In a thermocycler, anneal the smiFISH probes.

**Table:** smiFISH annealing program

Temperature	Time	Cycles
85°C	3 min	1
65°C	3 min	1
25°C	5 min	1

7. Store annealed smiFISH probes in the dark at −20°C.

***Note:*** It is possible that large aggregates of smiFISH probes may be present when imaging. If this occurs, refer to problem 2 in the troubleshooting section.

## Key resources table

**Table:** 

REAGENT or RESOURCE	SOURCE	IDENTIFIER
**Bacterial and virus strains**		

NEB 5-alpha competent *E. coli* (high efficiency)	New England Biolabs	C2987H

**Chemicals, peptides, and recombinant proteins**		

Kanamycin sulfate	Gibco	11815024
Tris base	Sigma-Aldrich	10708976001
Ethylenediaminetetraacetic acid (EDTA)	Promega	H5032
DEPC-treated water	Invitrogen	AM9920
NEBuffer 3	New England Biolabs	B7003S
UltraPure SSC, 20X	Thermo Fisher Scientific	15557044
Formamide	Thermo Fisher Scientific	17899
Dextran sulfate 50% solution	MilliporeSigma	S4030
PBS - Phosphate-buffered saline (10X) pH 7.4, RNase-free	Thermo Scientific	AM9625
Tween 20	Sigma-Aldrich	P9416
Sodium (meta)arsenite	Sigma-Aldrich	S7400-100G
RNase A	Thermo Scientific	EN0531
RNase I	Thermo Scientific	AM2294
Proteinase K, molecular biology grade	NEB	P8107S
Formaldehyde, 4% in PBS	Thermo Scientific	J60401.AK
DAPI (4′,6-diamidino-2-phenylindole)	Thermo Scientific	62248
VECTASHIELD	Vector Laboratories	H-1000-10

**Critical commercial assays**		

Lipofectamine 3000 Transfection ReagentGreen features	Thermo Scientific	L3000001
QIAprep Spin Miniprep Kit	QIAGEN	27104

**Deposited data**		

MATLAB scripts, FIJI macros, and representative images	Zenodo	10.5281/zenodo.14662082

**Experimental models: Cell lines**		

U-2 OS wt	ATCC	HTB-96
U-2 OS G3BP1-GFP	Paul Taylor Lab	Figley et al.^4^

**Oligonucleotides**		

FISH probes are listed in Table S1		

**Recombinant DNA**		

EGFP-NPM1	Addgene, Wang et al.^5^	17578
EGFP-β-Actin	Addgene, Rizzo et al.^6^	56421

**Software and algorithms**		

Oligostan	Tsanov et al.^2^	https://bitbucket.org/muellerflorian/fish_quant
Stellaris probe designer	Biosearch Technologies	https://www.biosearchtech.com/support/tools/design-software/stellaris-probe-designer
R	CRAN	https://cran.rstudio.com/
RStudio	Posit	https://posit.co/downloads/
Snapgene	SnapGene	https://www.snapgene.com/
A plasmid editor	Davis and Jorgensen	https://jorgensen.biology.utah.edu/wayned/ape/
MATLAB	MathWorks	https://www.mathworks.com/products/matlab.html
FISH-quant	Mueller et al.^12^	https://bitbucket.org/muellerflorian/fish_quant/src/master/
Image J	Schneider et al.	https://imagej.net/ij/
Fiji	Schneider et al.	https://imagej.net/software/fiji/downloads

**Other**		

Eppendorf Safe-Lock tubes 1.5 mL - microtube	Fisher Scientific	05-402-25
Round cover glass, #1.5 thickness, 12 mm	Thomas Scientific	1217N79
VWR micro cover glasses, rectangular	VWR	48404-467
Fisherbrand Economy Plain glass micro slides	Fisher Scientific	12-549-3
Nunc cell culture-treated multidishes	Thermo Scientific	142475
Fisherbrand High precision straight tapered ultra fine point Tweezers/Forceps	Fisher Scientific	12-000-122
Dulbecco’s modified Eagle’s medium (DMEM)	Invitrogen	12800082
Fetal bovine serum	Atlas Biologicals	F-05000-DR
Trypsin-EDTA solution	Invitrogen	25300120
Fisherbrand Petri dishes specialty (square)	Fisher Scientific	FB0875711A
Heathrow Scientific Parafilm M laboratory film	Fisher Scientific	PM999
Kimberly-Clark Professional Kimtech Science Kimwipes Delicate task wipers, 1-Ply	Fisher Scientific	34155
Silicone isolators	Grace Bio-Labs	JTR20R-0.5

## Materials and equipment

### FISH solutions

**Table:** Wash A (10 mL)

Reagent	Final concentration	Amount
SSC (20X)	2X	1 mL
Formamide	10%	1 mL
RNase free water	N/A	8 mL

SSC diluted in water can be stored indefinitely at 20°C–25°C before adding formamide. Formamide decomposes over time, so make an appropriate amount of Wash A and use as needed. Formamide is often packaged in amber bottles under argon gas to prevent decomposition into formic acid. After opening, formamide should be purged with nitrogen, aliquoted, and stored frozen for long-term storage or otherwise treated according to manufacturer’s instructions.

***Note:*** 3 mL of Wash A will be used per sample.

**CRITICAL:** Formamide is toxic. Use in a fume hood and dispose according to EH&S guidelines.

**Table:** Wash B (10 mL)

Reagent	Final concentration	Amount
SSC (20X)	2X	1 mL
RNase free water	N/A	9 mL

Wash B can be stored indefinitely.

***Note:*** 1 mL of Wash B will be used per sample.

**Table:** Hybridization Buffer (10 mL)

Reagent	Final concentration	Amount
SSC (20X)	2X	1 mL
Formamide	10%	1 mL
Dextran Sulfate (50%)	10%	2 mL
RNase free water	N/A	6 mL

Hybridization buffer can be stored indefinitely before adding formamide. Aliquot 450 μL or 900 μL into 1.5 mL tubes and store at −20°C. Add formamide immediately before using. Unmixed dextran sulfate solution can be stored at 20°C–25°C.

***Note:*** 50 μL of Hybridization will be used per sample.

***Alternatives:*** All FISH buffers can be purchased from Stellaris without formamide. High molecular weight, powdered dextran sulfate (Sigma cat #: D8906-5G) can be used instead of 50% solution, but it is challenging to dissolve.

### Other solutions

**Table:** PBSTween (50 mL)

Reagent	Final concentration	Amount
PBS (10X)	1X	5 mL
Tween-20 (20% in water)	0.2%	500 μL
RNase free water	N/A	44.5 mL

PBSTween can be stored indefinitely at 20°C–25°C.

## Step-by-step method details

### Cell culture and transfection of cells with fluorescent protein reporter

**Timing: 3 days**

This section describes how to seed and transfect U-2 OS cells with the fluorescent-tagged marker protein of interest. If a fluorescent protein was knocked in or otherwise expressed previously, transfection (Steps 3 and 4) can be skipped, and cells can be cultured normally before proceeding to Step 5 when they are at the desired confluency.

1. Sterilize 12 mm round coverslips by submerging them in 70% ethanol.
  a. Allow the residual ethanol to evaporate before rinsing with PBS.

***Note:*** Other coverslip sizes work so long as they can be manipulated in the 24-well plate.

2. Using sterile tweezers, rinse the coverslips in 1X PBS before moving the coverslips to the wells of a 24-well plate.
3. Seed cells on coverslips at a density such that they will be approximately 70% confluent the next day for transfection.

***Note:*** Investigators should test the optimal seeding density for their specific transfection.

4. After ∼16–24 h, transfect the plasmid encoding the fluorescently-tagged marker gene using the lipofectamine 3000 protocol: https://assets.thermofisher.com/TFS-Assets/LSG/manuals/lipofectamine3000_protocol.pdf.
5. Allow transfection to proceed for 16–48 h in a cell culture incubator.

***Note:*** Seeding density and transfection duration can be modified to maximize transfection efficiency.

***Note:*** Transfection can induce stress granules.^13^ If working with stress granules, ensure that enough time has passed to allow transfection-induced stress granules to resolve.

***Note:*** Alternative transfection reagents and protocols can be used.

***Note:*** Refer to the manufacturer’s instructions for troubleshooting and optimizing transfection.

### Permeabilization and enzyme treatment of cultured cells

**Timing: 30 min**

This section describes the protocol for permeabilizing U-2 OS cells with PBSTween and subsequently introducing degradative enzymes into the intracellular space.

6. If the organelle of interest requires induction, treat cells to form the assembly.
  a. E.g., to induce stress granule assembly, add 10 μL 50 mM (500 μM final) sodium arsenite to 1 mL DMEM to stress cells
7. Wash each sample three times with 1 mL 1X PBS.
  a. For temperature-sensitive organelles such as SGs, ensure the PBS is warmed to 37°C to prevent cold-shock-induced dissolution.
8. Aspirate the third PBS wash and add 1 mL 1X PBS containing 0.2% Tween 20.

***Note:*** Higher Tween concentrations improve enzyme penetrance at the cost of increased cell detachment in future steps.

9. Incubate samples in 0.2% PBSTween for 10 min.

***Note:*** Investigators can optimize permeabilization timing for their samples if needed.

***Note:*** If poor degradation of protein or RNA is observed upon imaging, refer to problem 3 in the troubleshooting section.

**Figure 1:**
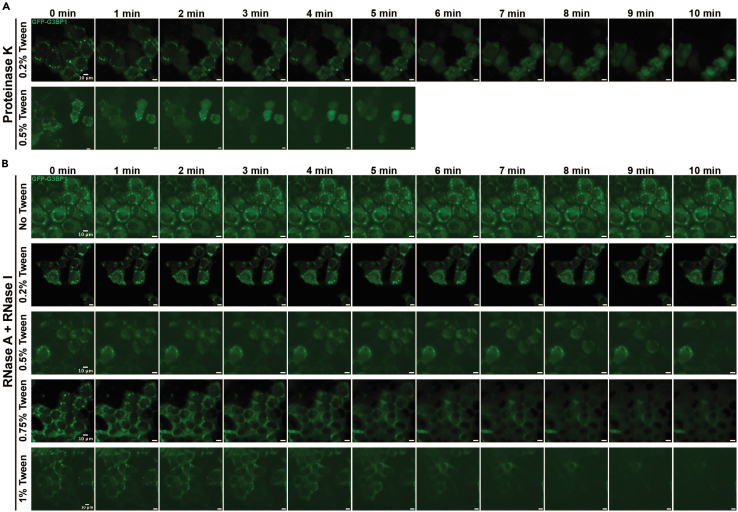
Titration of PBST with enzyme treatment (A) Time-course imaging of live cells with 0.02 U/μL of Proteinase K following a 10-min incubation in 0.2% or 0.5% PBSTween. Cells were treated with 500 μM of sodium arsenite for 1 h prior to treatment. G3BP1-GFP is shown in green. Scale bars are 10 μm. (B) Time-course imaging of live cells with 100 μg/μL of RNase A and 1 U/μL of RNase I following a 10-min incubation in PBST with varying concentrations of Tween 20. Cells were treated with 500 μM of sodium arsenite for 1 h prior to treatment. G3BP1-GFP is shown in green. Scale bars are 10 μm.

***Note:*** 100 μg/μL RNase A and 1 units/μL RNase I work well for degrading total cellular RNA.

***Note:*** 0.02 units/μL NEB Proteinase K, Molecular Biology Grade (P8107S) works well for degrading cellular proteins.

***Note:*** Investigators should titrate enzyme concentrations and test treatment durations for their organelle of interest.

***Note:*** Other degradative enzymes should work in this protocol as long as they function in residual Tween.

***Note:*** If it is necessary to wash with PBS to remove Tween for enzyme function, wash once in 1 mL PBS, but do not let the cells sit in PBS for long as they quickly become less permeable upon removal of Tween.

10. Gently aspirate the PBSTween from the samples.
11. Add 100 μL of PBS-enzyme solution to the samples and incubate for 5–10 min (Figure 1).

**CRITICAL:** From this point forward, pipette solutions very slowly and gently. Cells become prone to detaching from coverslips/wells following detergent permeabilization and are **extremely** prone to detaching following protease treatments. Leaving some solution (∼100–200 μL) in the well when pipetting solutions off and adding solutions slowly to the wall of wells helps reduce cell loss during washes. If there are very few cells left for imaging at the end of the protocol, refer to problem 4 in the troubleshooting section.

12. Add 500 μL 4% 20°C–25°C formaldehyde directly to the PBS-enzyme-containing sample wells.
13. Fix in formaldehyde for 15 min at 20°C–25°C.

### Performing smFISH or smiFISH on permeabilized, enzyme-treated samples

**Timing: 1 day**

This section describes the modifications to standard RNA FISH protocols required to visualize RNAs in permeabilized, enzyme-treated cells.

**Reminder:** Pipette as gently as possible throughout the remainder of this protocol. Cells are still prone to detaching after fixation.

14. Aspirate the formaldehyde solution from the sample wells.

**CRITICAL:** Formaldehyde is toxic. Dispose of formaldehyde-contaminated waste according to EH&S guidelines.

15. Wash samples gently in 1 mL 1X PBS three times.
16. Aspirate the remaining PBS and replace it with 1 mL Wash A solution.

**CRITICAL:** Formamide is toxic. Dispose of formamide-contaminated waste according to EH&S guidelines.

***Note:*** It is okay to have some residual PBS when adding Wash A to allow for gentle addition of Wash A. Diluting the Wash A solution ∼10% has not affected FISH experiments.

17. Incubate samples in Wash A for 5 min.
18. While incubating, add probes to the hybridization solution.
  a. Prepare a 50 μL hybridization solution for each sample to be stained.

***Note:*** Typically, 1:100 dilutions of smFISH probe stocks or annealed smiFISH probes in RNase free water work well. Investigators should determine the optimal probe dilution factor for their target RNAs.

19. Still while incubating, prepare a FISH hybridization chamber.
  a. Cut a paper towel and a piece of parafilm to fit in a square petri dish.
  b. Place the cut paper in the tray and wet the paper, pouring off any excess water.
  c. Place the parafilm on the wetted towel with the parafilm side up. Do not remove the wax paper.
  d. Flatten the parafilm to prevent coverslips from drifting.
  e. Pipette 50 μL droplets of the probe containing hybridization solution on the parafilm for each sample to be hybridized.
  f. Add sample labels near each droplet before moving samples.

***Note:*** Once removed from the 24-well plate, the coverslips will have no identifying features.

***Note:*** Square petri dishes can be replaced by any chamber that will keep the samples sealed and humid at 37°C–16 h. For example, a pipette tip box with water in the reservoir and parafilm on the tip holding tray can also work.

20. Using fine-point tweezers, gently remove the coverslips from the Wash A solution.

***Note:*** Grasping coverslips is easier if the Wash A solution is **not** aspirated. Removing the Wash A completely can detach more cells and will adhere the coverslip to the well through surface tension.

***Note:*** If removing coverslips from wells is challenging, slightly lift the coverslip by pressing it against the side of the well with tweezers before sticking a 10 μL pipette tip under the coverslip to prevent it from falling flat. The tweezers can then be used to grab the coverslip more easily.

21. Gently place the coverslip cell-side down into the probe-containing hybridization solution.
  a. Do not press the coverslip into the hybridization solution. The coverslip should float on top of the solution.

***Note:*** The hybridization solution should completely cover the surface of the coverslip.

22. Seal the hybridization chamber with parafilm or place it in a Ziplock bag to prevent evaporation.
23. Place the hybridization chamber in a dark 37°C incubator for 16–24 h.
24. After ∼16 h hybridization, aspirate the Wash A solution in the original 24-well plate.
25. Add 1 mL Wash A solution to each well.
26. Gently remove the coverslips from the hybridization chamber and slowly place them cell-side up in the Wash A solution.
27. Incubate the samples at 37°C in the dark for 30 min.
28. While incubating in Wash A, add 5 μL/mL of 1 μg/mL DAPI to the remaining Wash A solution.
29. Gently aspirate the Wash A solution and replace it with 1 mL Wash A containing DAPI.
30. Incubate the samples at 37°C in the dark for 30 min.
31. Aspirate the Wash A solution and replace it with 1 mL of the Wash B solution.
32. Incubate at 20°C–25°C for 5 min.

### Slide preparation and imaging

**Timing: 1–4 h**

This section describes the process for preparing slides and visualizing enzyme-treated cells. It also presents additional considerations for imaging to ensure robust data is acquired.

33. Prepare slides for coverslip mounting (Figure 2).
  a. Label slides according to sample identity.
  b. Place a 24 × 30 mm rectangular coverslip in the middle and perpendicular to the slide.

**Figure 2:**
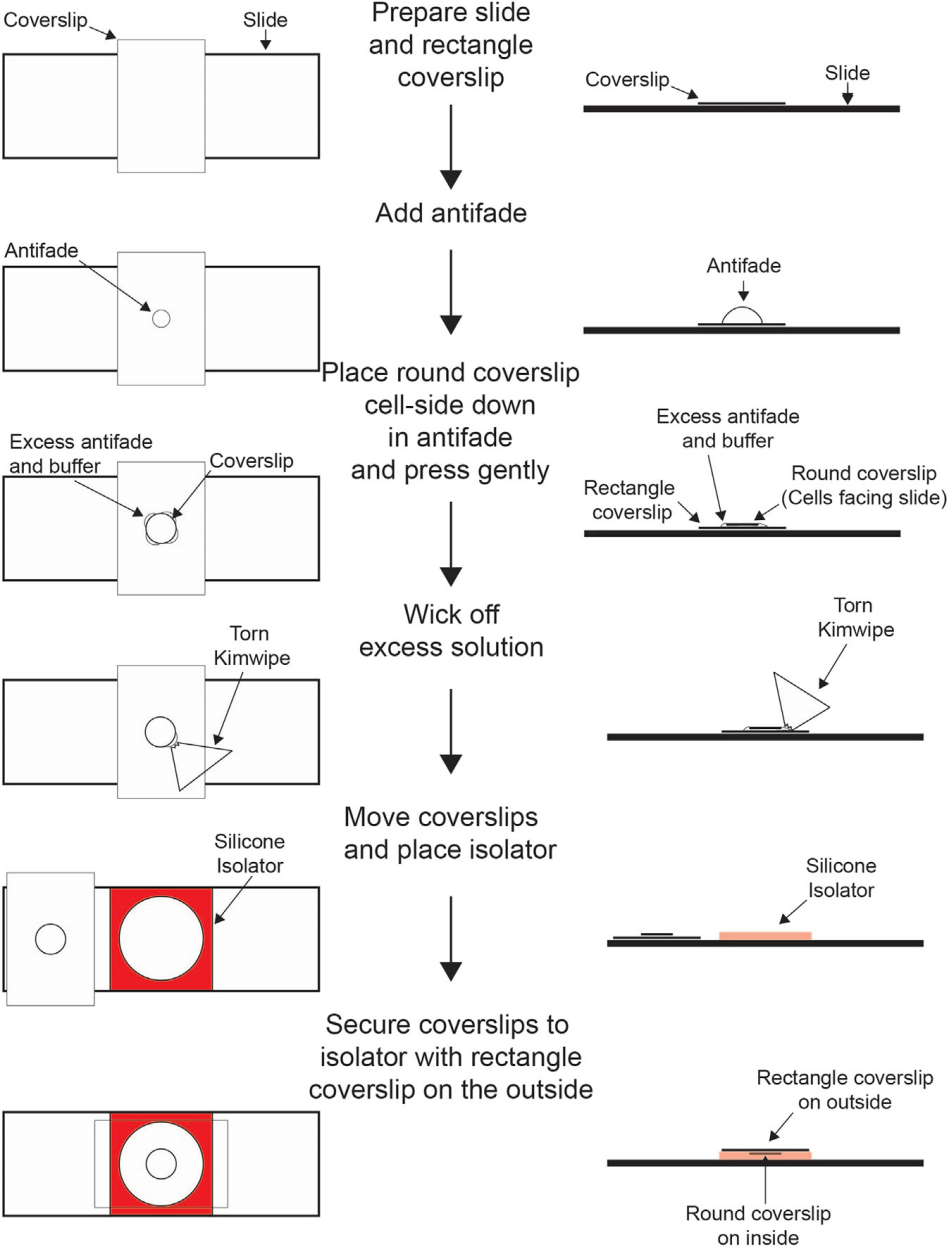
Slide making schematic A visual depiction of the protocol for slide assembly using the “coverslip sandwich” method.
34. Place 1 μL VECTASHIELD mounting medium in the center of each rectangular coverslip.
35. Using fine-point tweezers, gently remove the coverslips from the Wash B solution.
36. Place the round coverslip with cells face down in the VECTASHIELD solution.
37. Very gently press down on the coverslip to expel excess solution from between the coverslips.

**CRITICAL:** Do not allow the coverslips to slide side-to-side when pressing on them. The shear force can completely remove the weakly attached cells from the coverslip. If pressing on the coverslips proves challenging, samples can be wicked more during the next step.

38. Using a torn Kimwipe, wick away excess solution from between the coverslips.

***Note:*** Tearing the Kimwipe speeds up wicking.

39. Move the coverslip “sandwich” to the side of the slide.
40. Adhere a Grace Bio-labs Silicone Isolator (JTR20R-0.5) to the center of the slide.
41. Place the coverslip sandwich so that the smaller round coverslip faces the slide and the rectangular coverslip faces the outside and parallel to the slide.
42. Gently press the rectangular coverslip directly over the silicone isolator to adhere the coverslip to the slide.
  a. Exercise caution when adhering coverslips, as they can crack or shatter during this step if pressed too hard or not directly over the isolator.
43. Store slides in the dark until imaging.
  a. Image slides on the day of preparation.
44. Place the sample on a microscope.

***Note:*** Epifluorescent or confocal microscopes work for imaging FISH samples.

***Note:*** 60X objectives or above are recommended for imaging FISH spots for downstream quantification, although lower magnifications may work in some cases.

45. Set lamp/laser powers to the appropriate levels to image the sample.

***Note:*** Investigators should identify the correct imaging settings for their proteins and RNAs of interest.

**CRITICAL:** If the images will be quantified, use identical imaging settings across sample conditions and replicates to compare fluorescent intensities fairly.

**Figure 3:**
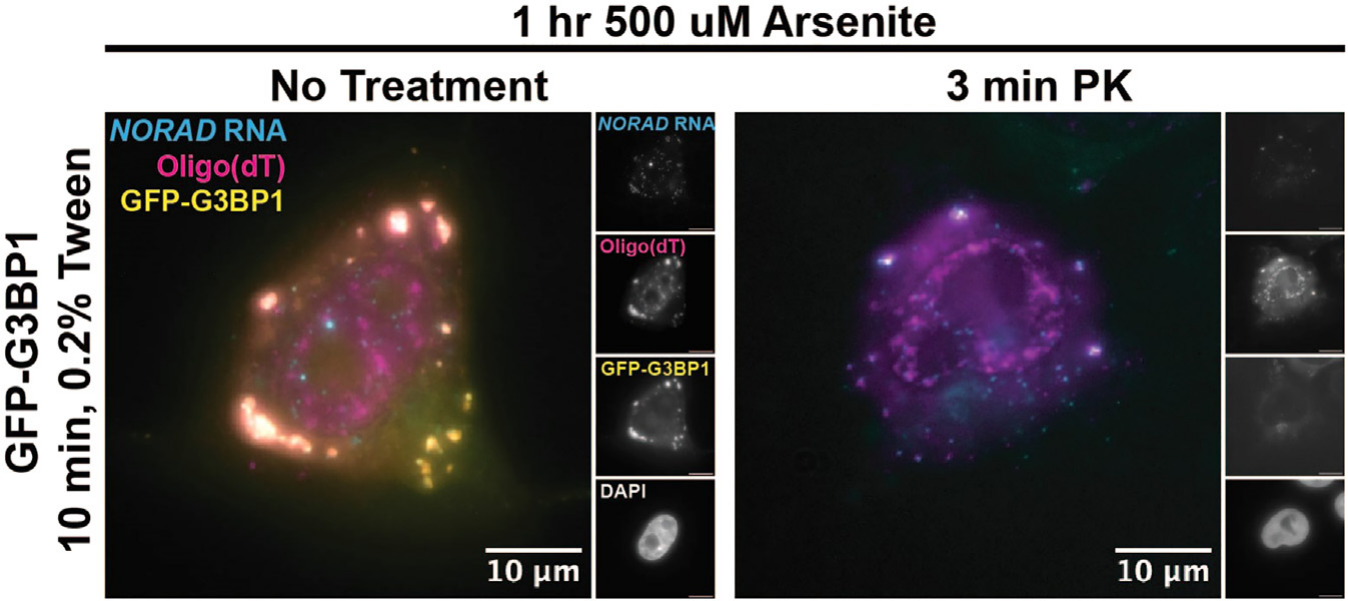
Proteinase K treatment of stress granules Stress granules were induced in G3BP1-GFP expressing U-2 OS cells by incubating with 500 μM of sodium arsenite for 1 h. Cells were then subjected to the permeabilization, enzyme treatment, and smFISH protocol using 0.2% Tween for permeabilization and a 3 min treatment with 0.02 U/ul of Proteinase K. Following protein degradation, G3BP1-GFP (yellow) signal is lost whereas the RNA signal of *NORAD* (cyan) and *PolyA* (magenta) are retained in granules. Scale bars are 10 μm.

**Figure 4:**
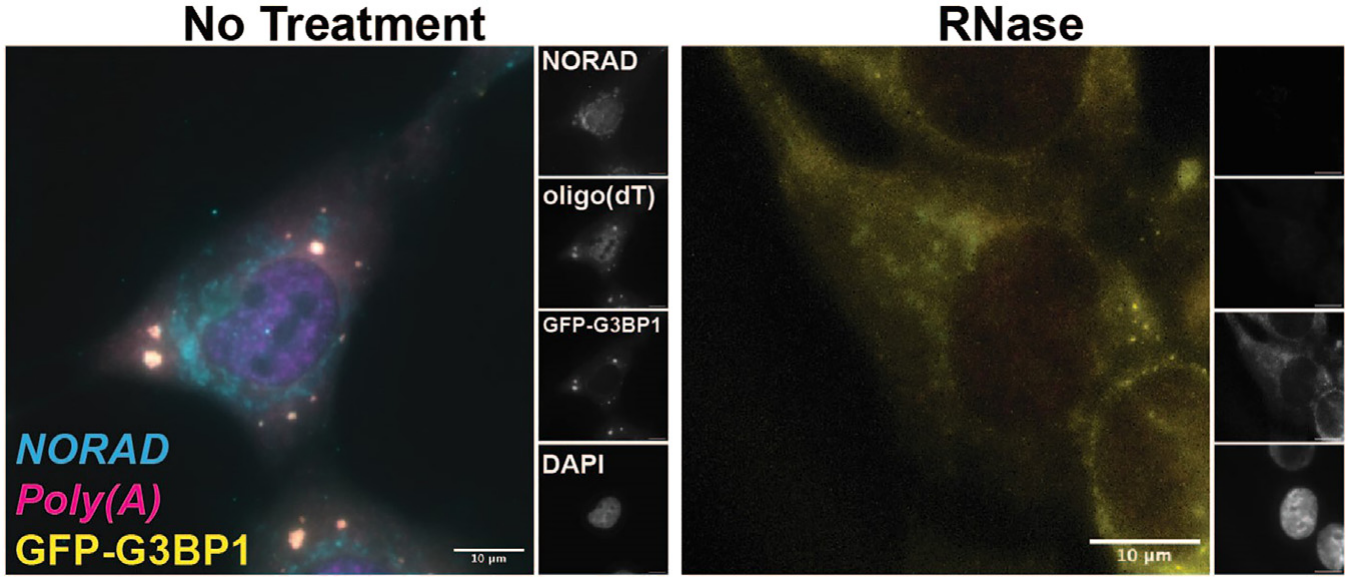
RNase treatment of stress granules Stress granules were induced in G3BP1-GFP expressing U-2 OS cells by incubating with 500 μM of sodium arsenite for 1 h. Cells were then subjected to the permeabilization, enzyme treatment, and smFISH protocol using 0.2% Tween for permeabilization and 100 μg/μL of RNase A and 1 U/μL RNase I. Following RNA degradation, both RNA (*NORAD* RNA in cyan, *PolyA* RNA in magenta) and protein (yellow) signal is lost from granules. Scale bars are 10 μm.

**Figure 5:**
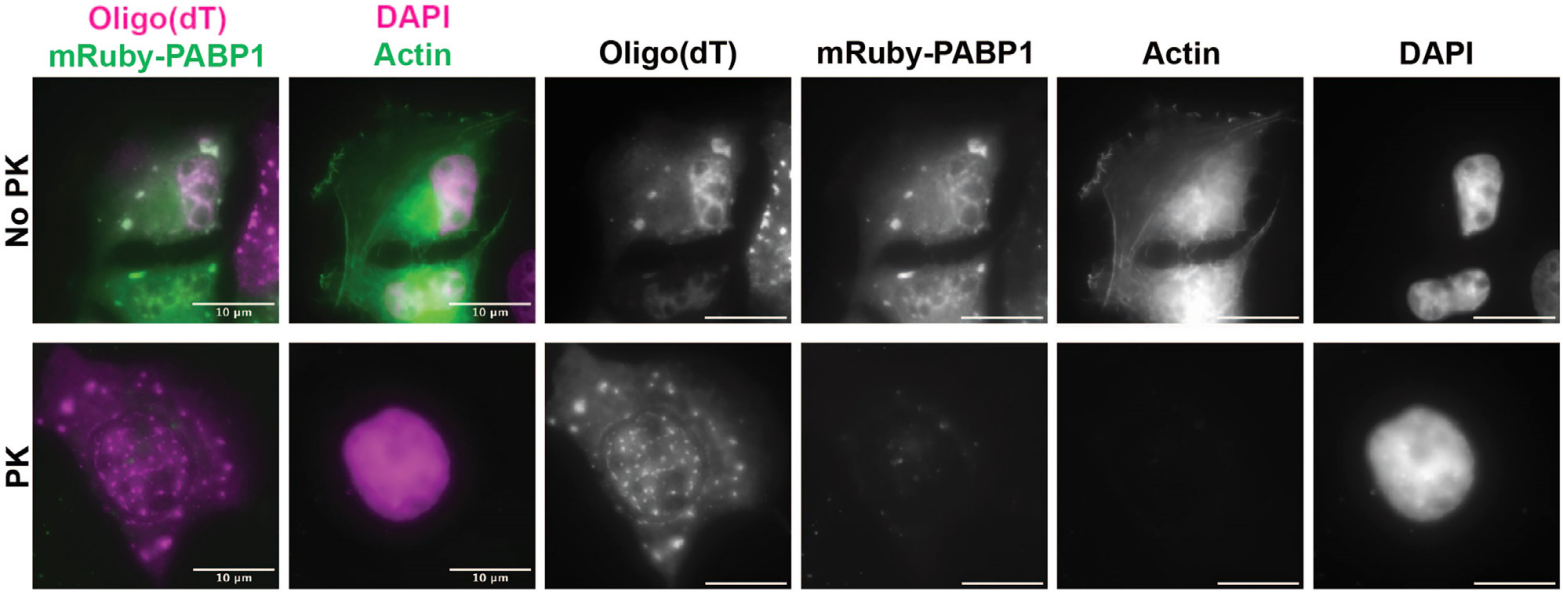
Proteinase K treatment of actin and alternative stress granule marker, PABP1 mRuby-PABP1 expressing U-2 OS cells were transfected to express eGFP-B-actin two days prior to treatment. Stress granules were induced by incubating with 500 μM of sodium arsenite for 1 h. Cells were then subjected to the permeabilization, enzyme treatment, and smFISH protocol using 0.2% Tween for permeabilization and a 3 min treatment with 0.02 U/ul of Proteinase K. Following protein degradation, mRuby-PABP1 (green, first column) signal is lost whereas the RNA signal of *PolyA* (magenta, first column) is retained in granules. Proteinase K treatment similarly degrades the eGFP-Actin (green, second column) signal, demonstrating that the treatment is not selective for particular protein targets. Scale bars are 10 μm.

**Figure 6:**
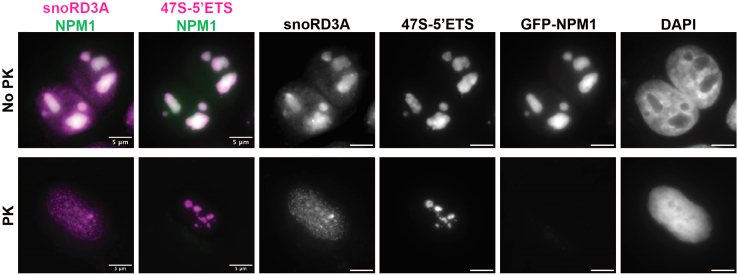
Proteinase K treatment of nucleoli U-2 OS cells were transfected with eGFP-NPM1 to label nucleoli. Two days later cells were subjected to the permeabilization, enzyme treatment, and smFISH protocol using 0.2% Tween for permeabilization and a 3 min treatment with 0.02 U/ul of Proteinase K. Following protein degradation, eGFP-NPM1 (green) signal is lost. Notably, *snoRD3A* RNA (magenta, first column) disperses whereas *47S-5′ETS* RNA (magenta, second column) is retained in nucleoli after protein degradation. This suggests that some RNAs can migrate in nucleoli, and others are trapped in RNA networks. Scale bars are 5 μm.

46. Identify cells for imaging.
  a. Look for cells with morphology that is not obviously perturbed.
    ***Note:*** Permeabilization and enzyme treatment, particularly with proteinase, can damage cells. Imaging damaged cells can confound results.
    ***Note:*** Some things to look out for are: 1) Intact nuclei. Protease treatment should not disrupt the shape of the nuclei nor the presence of nucleolar dim spots as visualized by DAPI staining. 2) The presence of cytoplasm. If cells are over-digested, it is possible to find cell-free nuclei, which are not suitable for imaging. 3) Normal cell shape. For instance, the characteristic cell protrusions of U-2 OS cells should remain following protease treatment. The latter two morphological characteristics are visible under bright-field or DIC microscopy if cytoplasmic fluorescence is not present.
  b. In experiments treating cells with protease, investigators should ensure they are imaging cells containing the fluorescent protein marker of interest.
    ***Note:*** If not every cell expresses the fluorescent protein, i.e., if the cells were transfected for protein expression, controls must be in place to ensure robust results.
    ***Note:*** The preferred method for validating transfection is probing for the RNA expressed from the transfected plasmid by FISH to provide an internal control for transfection on the cell-to-cell level.
    i. If it is not possible to use FISH as an internal control, calculate transfection efficiency using a non-enzyme-treated control sample and image sufficient cells in the protease-treated sample to guarantee that a significant proportion was transfected.
  c. In experiments where cells are treated with RNases, investigators should ensure that the FISH staining was successful by comparing it to a non-RNase-treated control.
    ***Note:*** Permeabilization and enzyme treatment are typically not 100 % penetrant, so some cells in the RNase-treated samples should still have normal RNA signals.
47. Image the complete depth of the cells of interest using z-stacks. Several examples of images generated with this protocol are shown in Figures 3, 4, 5, and 6.
  a. For effective quantification of diffraction-limited FISH spots, it is recommended to image at the axial resolution limit for the objective being used.
  b. Imaging to the point of being slightly out of focus on either axial end of the acquisition is recommended to ensure that the entire sample is imaged and no FISH spots are missed.
  c. If samples appear to drift during imaging, refer to problem 5 in the troubleshooting section.
  d. If FISH spots are not visible during imaging, refer to problem 6 in the troubleshooting section.

## Expected outcomes

Here, we present a protocol that allows degradative enzymes into the intracellular space of living cells. Subsequently, performing RNA FISH on enzyme-treated samples allows users to determine whether protein, RNA, or both are required for the persistence of membraneless organelles. Using this protocol, we have demonstrated that protein is dispensable for the ultrastructural organization of both stress granules and the nucleolus in U-2 OS cells, while RNA is essential for their continued persistence.^1^ Other investigators will similarly be able to assess whether particular cellular components are essential or dispensable for assembly organization by testing if degrading RNA or proteins disperses cellular assemblies.

## Quantification and statistical analysis

**Timing: ∼1–3 days**

There are three useful analysis pipelines for analyzing data produced from this permeabilization and enzyme treatment protocol. The first pipeline quantifies the total number of RNAs identified in a cell and determines the fraction localized within assemblies. This analysis requires that individual RNA spots can be resolved or deconvolved using a Gaussian mixture model which is, therefore, most useful when probing for lowly to moderately abundant transcripts, as for most mRNAs. The second pipeline determines the average intensity of assemblies over a distance. It is suited to analyzing non-discrete signals, such as highly abundant RNAs (e.g., polyA, *ETS-1*) and protein fluorescence. The final analysis determines the average maximum intensity of components within a region of interest, which can be applied to any signal.

### Analysis 1: Determination of RNA count and fraction in assemblies

1. Identify individual FISH spots using FISH-quant.^12^^,^^14^
  ***Note:*** We provide instructions for using the MATLAB implementation of FISH-quant here. The online implementation can also be used at https://fish-quant.github.io/. Alternatively, the recently developed dNEMO can be used to identify FISH spots.^15^ However, because the add-ons used to deconvolve granular RNAs and calculate overlap with fluorescent markers were developed using FISH-quant, these scripts may need editing to be compatible with the output of dNEMO.
  a. Download and install FISH-quant from https://bitbucket.org/muellerflorian/fish_quant/src/master/ following the instructions here: https://bitbucket.org/muellerflorian/fish_quant/src/master/Documentation/FISH_QUANT_v3.pdf.
    ***Note:*** In addition to the suggested toolboxes, the Signal Processing toolbox is required.
  b. Download the supplemental FISH-quant Matlab scripts and FIJI macros from https://zenodo.org/records/14662082. Save the MALAB scripts “Wrapper_colocPbodies_v1.m,” “ColocPbodies_loop_v1.m,” and “colocPbodies_v3.m” under FISHquant/toolbox/.
  c. Prepare images for analysis by cropping to a single cell of interest, splitting multicolor images, and saving them as individual stacks in FIJI.
  d. Save each FISH channel in a unique subfolder titled “C#” where # corresponds to the channel number.
    ***Note:*** For example, if the far-red channel was imaged first, save it under the folder “C1” after splitting the image.
  e. Open FISH-quant and identify spots using the standard FISH-quant analysis pipeline.
    i. Click “Modify” under “Experimental parameters” and adjust the pixel-size xy, pixel-size z, refractive index, numeric aperture, excitation wavelength, and microscope to reflect experimental parameters.
    ii. Click “Load image” and import the FISH channel to be quantified.
    iii. Click “Define outlines.”
    iv. Click “New cell” in the popup window and outline the cell.
    v. Click “[FQ] Outline” and save the outline in the folder the image is stored.
    vi. Close the “comment” popup and the outline designer.
    vii. Click “Filter”. The standard settings are typically satisfactory.
    viii. Click “Detect” and enter test values for pre-detection.
      ***Note:*** The output curve should have a distinct plateau or inflection point on the “Number of detected spots” axis, indicating the average intensity of an individual FISH spot.
    ix. Click “Perform detection.”
    x. Click “Show detected spots” and verify that the chosen threshold detects the appropriate spots.
      If FISH-quant fails to identify many spots and simultaneously detects spots where there are no RNA foci, refer to problem 7 in the troubleshooting section.
      If FISH-quant struggles to identify closely grouped RNA spots, refer to problem 8 in the troubleshooting section.
    xi. Close the spot detector.
    xii. When satisfied with the chosen threshold, click “Apply threshold to all cells.”
    xiii. In the “[FQ] main” dropdown, go to “Save” and then “Detection settings,” and save the detection settings under the same folder as the image and outline.
    xiv. Repeat Steps i-xiii for each FISH channel to be analyzed.
2. Deconvolve RNA assemblies using a Gaussian Mixture Model.
  a. Open the “WRAPPER_analyze_smFISH__EXP_v1.m” analysis script under FISHquant/locFISH/ in MATLAB.
  b. Click “Run” and select the parent folder containing all FISHquant analysis to run the GMM as a batch process.

**CRITICAL:** The Gaussian Mixture Model can only be applied to samples with a substantial population of discrete single-molecule spots in addition to large assemblies of multiple molecules. For non-discrete FISH signals, such as PolyA, use analysis 2.

3. Generate a multipoint ROI of detected FISH spots.
  a. Open the C#-FILE-NAME_res_GMM.txt analysis file in Excel.
    ***Note:*** The file is stored in the FISH-quant analysis folder under “C#” and “results_GMM.” “C#” represents the channel folders made in 1c, and “FILE-NAME” is the user’s microscopy image file name.
  b. Divide the Pos_Y and Pos_X columns by the pixel size present in the same file as “Pix-XY.”
    ***Note:*** This converts the relative FISH spot coordinates generated in FISHquant to usable coordinates in FIJI.
  c. Save the new coordinates as a .csv file with column A containing X values and column B containing Y values. Use “X” and “Y” as column headers.
  d. With the microscopy image being analyzed open, make a maximum intensity projection by clicking “Image,” then “Stacks,” then “Z project,” and selecting the max intensity option.
  e. Use the “Spot_coordinate.ijm” FIJI macro to import a multipoint ROI of the detected FISH spots.
    ***Note:*** If done correctly, the ROIs should overlap with the FISH spots in the image.
  f. Hit the “t” key to save the multipoint ROI in the ROI manager.
  g. Select the ROI set and save it by clicking “More” and then “Save” in the ROI Manager.
4. Generate masks of the organelle of interest.
  a. Make sure the same multichannel, z-projected image is open in FIJI.
  b. Smooth the image by selecting “Process,” then “Filters,” then “Gaussian Blur.”
    i. Determine an appropriate level of smoothing that allows for consistent masking without generating many low-pixel-number masks.
      ***Note:*** Poor smoothing will become apparent in Step d and f when generating selections.
  c. Using a channel that is consistent between control and enzyme treatment conditions, click “Image,” then “Adjust,” then “Threshold,” and set the optimal threshold for masking the organelle of interest.
    ***Note:*** If there is not a clear threshold that works well, test different thresholding modes by clicking the panel that is titled “Default.” If this still does not work well, it is reasonable to create a threshold using deconvolved images to improve masking. However, the final quantification **must** overlay these masks on raw (not deconvolved) images. Alternatively, masks can be generated manually.
  d. Create a selection by clicking “Edit,” then “Selection,” then “Create Selection.”
  e. Add the selection to the ROI manager by hitting the “t” key.
  f. Select the ROI and save it by clicking “More” then “Save” in the ROI Manager.
  g. Close the smoothed z-projection.
5. Quantify the number of FISH spots overlapping with the organelle mask.
  a. Open the same raw image and make a new, unblurred z-projection.
  b. If the ROIs are no longer in the ROI Manager, reload them with the FISH spots as the first ROI and the organelle mask as the second.
  c. Use the Spot_overlap.ijm FIJI macro to determine how many FISH spots lie within the organelle mask.
    ***Note:*** The total number of FISH spots and the number within the organelle mask will be printed in the FIJI Log.
  d. Save the results.
6. Plot the results.
  a. The data can be plotted using the investigators’ preferred software and plot styles.

**Figure 7:**
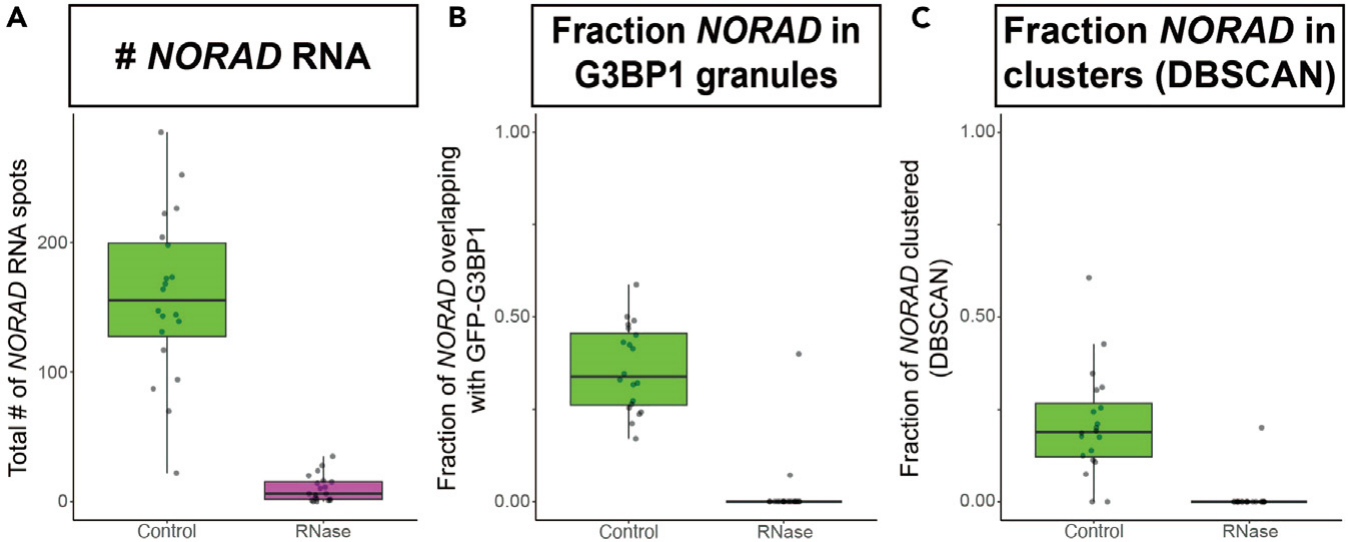
Quantification approach 1: FISH-quant, mask overlap, and DBSCAN (A) The total number of *NORAD* RNA spots present with and without RNase treatment were quantified using approach #1. FISH spots were first identified using FISH-quant. Assemblies of multiple RNAs were then deconvolved using a Gaussian Mixture Model to determine the number of RNAs within each assembly. (B) RNA spots identified in (A) were compared with manually generated masks of G3BP1-GFP granules to determine the fraction of *NORAD* RNA within stress granules. (C) The granularity of *NORAD* RNA was determined independently of a protein marker using DBSCAN. DBSCAN was run using a minimum of 5 RNAs to define an RNA cluster with an epsilon distance of 750 between each RNA. *n* = 20 cells from 4 biological replicates for each plot.

***Note:*** Using ggplot2 in R to present the data as box plots is an easy and effective way to compare control and enzyme treatments. See examples in Figure 7.

7. **Optional step:** Quantify RNA granularity using DBSCAN. See example in Figure 7C.
  ***Note:*** The remaining steps in this analysis pipeline use a DBSCAN algorithm to determine whether individual RNA molecules are in proximity. This analysis can be a useful proxy for RNA granularity when no good marker exists for the organization of an enzyme-treated organelle.
  a. Open the MATLAB script “WRAPPER_colocPbodies_v1.m” stored under FISHquant/Toolbox/.
  b. Replace the folder in line 23 with the path to the parent folder of the specific image to be analyzed, i.e., the folder above the “C#” set of folders.
  c. Replace the folder in line 30 with the path to the folder with the FISH channel to be analyzed, i.e., the “C#” folder.
  d. Ensure the variables in lines 39, 45, and 49 reflect the FISH channel file prefix, the GMM file suffix, and the fluorescent protein channel file prefix, respectively.
  e. Modify the param.epsilon and param.MinPts variables to suit the density of RNAs in clusters.
    ***Note:*** The standard parameters typically work well.
    ***Note:*** The epsilon variable measures how far apart RNAs can be to be considered within a cluster.
    ***Note:*** The MinPts variable defines the minimum number of neighboring RNAs that comprise a cluster.
    ***Note:*** If MATLAB finds the undefined variable “err,” it is necessary to download the ghostscript package. This can be found at http://www.ghostscript.com for Windows computers and http://pages.uoregon.edu/kock/ for Mac computers. Restart MATLAB and the wrapper in order to allow the new changes to be used.
  f. Run the script.
    ***Note:*** The command window output will show the total number of RNAs, the number of RNAs in clusters, and the number of clusters.
    ***Note:*** If the size of individual clusters is needed for analysis, this info is saved in the new folder starting with “db-” in the “CoLoc__results.mat” file.
    ***Note:*** Opening this file in MATLAB and clicking on “tbl” will produce a spreadsheet of granule results. The first row is the number of singlet RNAs, and every following row is an individual cluster. The second column represents the number of RNAs in each cluster, and the third column represents the percentage of RNAs in each cluster.

### Analysis 2: Determination of fluorescence intensities across an assembly

**Figure 8:**
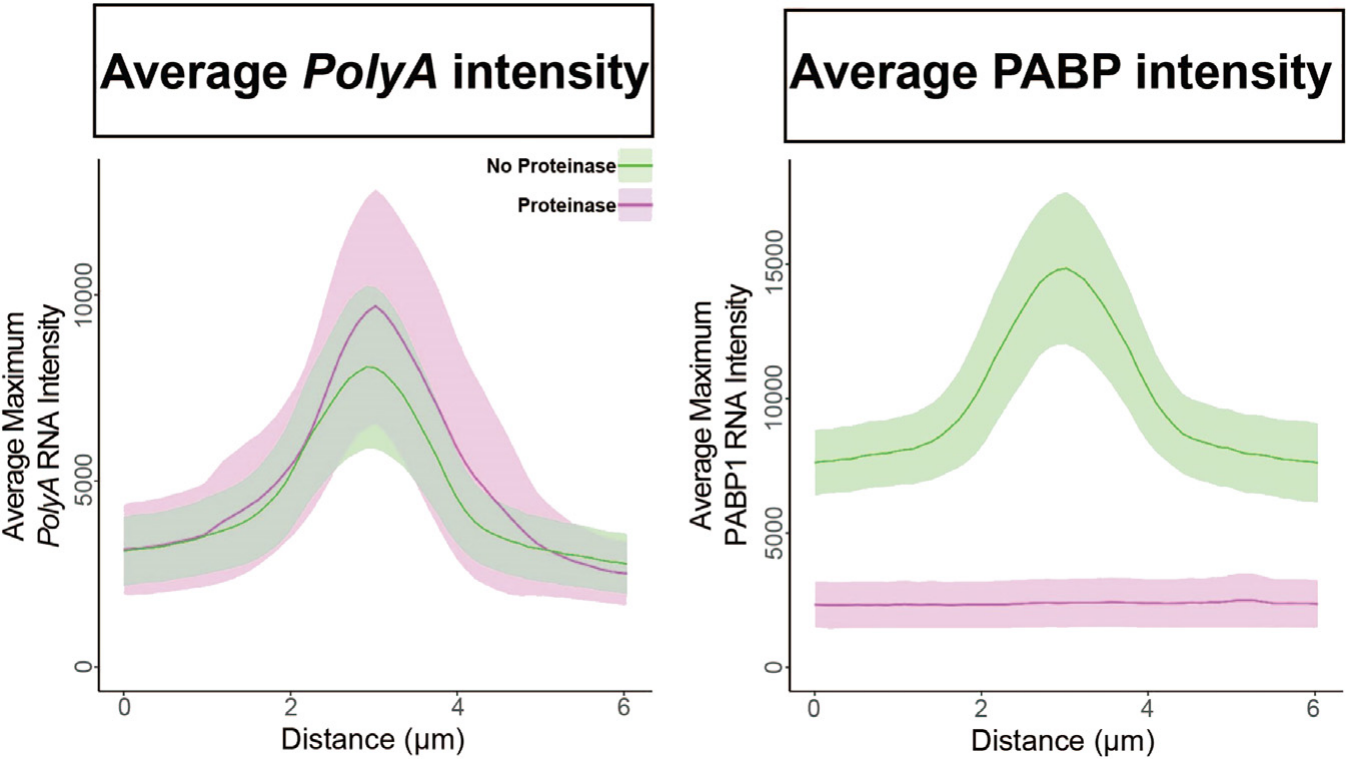
Quantification approach 2: Averaged line scans The maximum intensity of *PolyA* RNA (left) and PABP1 (right) was measured across a 6-μm distance going through a stress granule. *n* = 20 line scans from 3 biological replicates were centered around the maximum intensity of the scan and averaged. The line represents the mean maximum intensity, and the shaded ribbon represents the 95% confidence interval.

***Note:*** Using ggplot2 in R to present the data as line plots with ribbons for error bars is an easy and effective way to compare control and enzyme treatments.

***Note:*** Normalizing the intensities to the average maximum intensity in control samples can simplify the interpretation of changes between enzyme treatments.

***Note:*** Using 95% confidence intervals as the error bar ribbons makes a visual assessment of significance straightforward.

8. Perform line scans across assemblies.
  a. Open the image to be quantified in FIJI.
  b. Make a maximum intensity projection by clicking “Image,” then “Stacks,” then “Z project,” and selecting the max-intensity option.
  c. Select the “∗straight∗” line tool in the FIJI toolbar.
  d. Draw a line through the assembly of interest, making the line extend some distance beyond the assembly on each side.
    ***Note:*** It is recommended to save the line used for analysis as an ROI for posterity.
  e. Press the “k” key to measure the line intensity profile.
  f. In the window that pops up, select “Data” and “Save Data” to save the line profile data.
  g. Repeat Steps a-f on multiple assemblies in multiple cells until sufficient data has been collected.
9. Align line profiles from different assemblies.
  a. Open the line profile data spreadsheets.
  b. Determine the maximum value in the scan to set a center point.
  c. Subset the line profile data to include only a set distance on either side of the maximum intensity point.
    ***Note:*** For stress granules, 3 μm on either side of the maximum provides enough distance to span typical stress granules. Determine a suitable distance for the assembly of interest.
  d. Combine data across multiple scans by averaging the intensity at each distance. For example, if a 6 μM-long line profile is measured, average all values at distances 0 μM, 1 μM, 2 μM…etc.
10. Plot averaged line profiles. See examples in Figure 8.
  a. The data can be plotted using investigators’ preferred software and plot styles.

### Analysis 3: Quantify average maximum intensities of discrete FISH spots, RNA assemblies, and fluorescently labeled organelles

**Figure 9:**
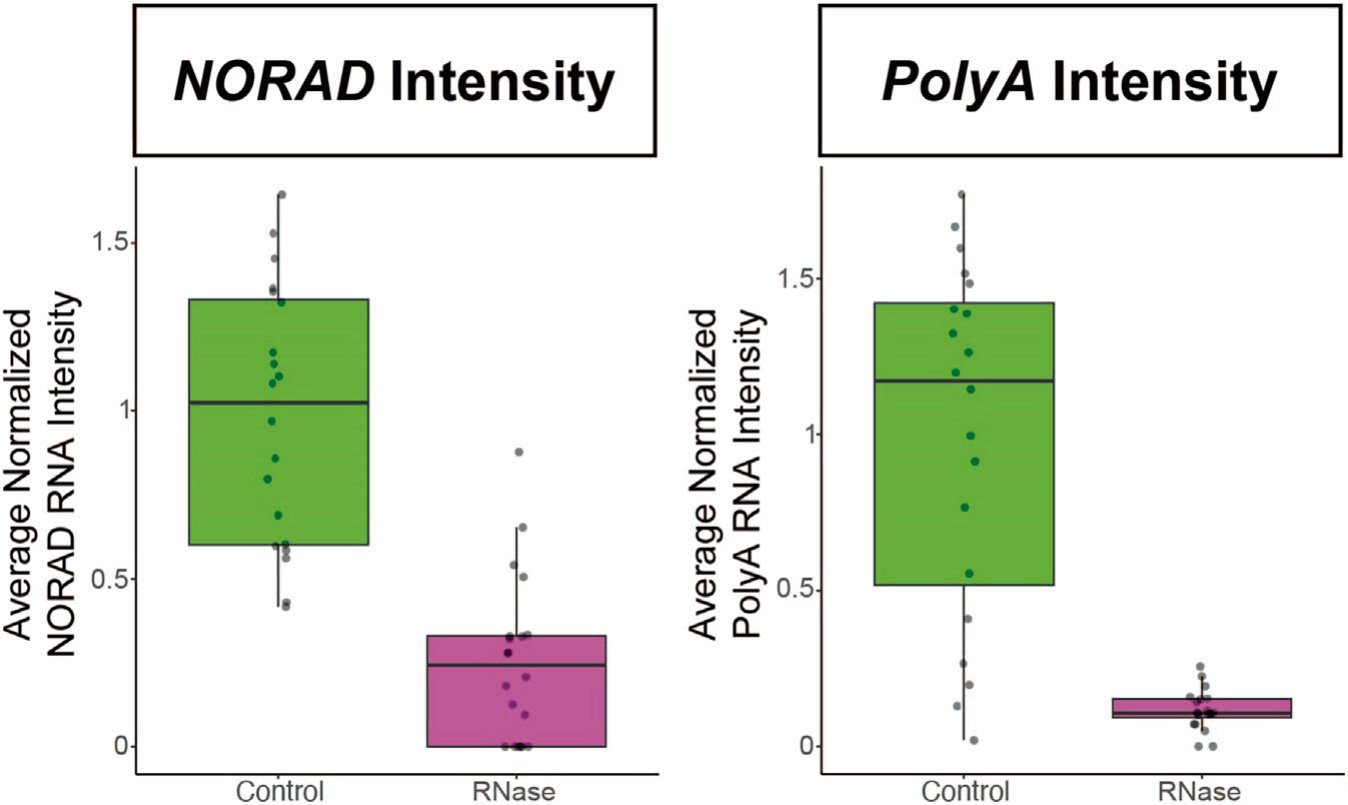
Quantification approach 3: Measuring total intensity Using FISH spot and *polyA* assembly masks generated in Figure 7, total intensities of RNA were measured between no enzyme and RNase treatment conditions.

11. Determine the intensity of discrete FISH spots.
  a. Open the image to be quantified in FIJI.
  b. Make a maximum intensity projection by clicking “Image,” then “Stacks,” then “Z project,” and selecting the max-intensity option.
  c. Open the multipoint ROI set generated using FISHquant and FIJI in Step 3g.
  d. In the ROI Manager, click “Measure.”
  e. Save the “Results” table popup.
  f. Repeat Steps a-e for all images to be quantified.
12. Determine the intensity of RNA assemblies and fluorescently labeled organelles.
  a. Open the image to be quantified in FIJI.
  b. Make a maximum intensity projection by clicking “Image,” then “Stacks,” then “Z project,” and selecting the max-intensity option.
  c. Open the ROI generated using FIJI in Step 4f.
  d. Split the selection into individual ROIs by selecting “More” and “Split” in the ROI Manager.
    i. Delete the first ROI, which is the combined selection.
    ii. Make sure there are a reasonable number of ROIs at this stage. If there are many more than expected, it is likely due to poor image smoothing.
    iii. If desired, manually remove any aberrant ROIs by selecting them and clicking “Delete” in the ROI Manager.
  e. Select all of the split ROIs.
  f. In the ROI Manager, click “Measure.”
  g. Save the “Results” table popup.
  h. Repeat Steps a-g for all images to be quantified.
13. Determine the background intensity.
  a. Open the image to be quantified in FIJI.
  b. Make a maximum intensity projection by clicking “Image,” then “Stacks,” then “Z project,” and selecting the max-intensity option.
  c. Create multiple selections of regions in the cell that do not have FISH spots or protein assemblies.
    ***Note:*** Three one square micron background regions is typically sufficient to get good representation of background fluorescence.
  d. In the ROI Manager, click “Measure.”
  e. Save the “Results” table popup.
  f. Repeat Steps d and e in the other channels to be analyzed.
  g. Repeat Steps a-f for all images to be quantified.
14. Plot average maximum intensities. See examples in Figure 9.
  a. Subtract the average intensity of the background signals from the FISH spot and fluorescent protein signals.
  b. The data can be plotted using the investigators’ preferred software and plot styles. Using ggplot2 in R to present the data as boxplots is an easy and effective way to compare control and enzyme treatments.

## Limitations

There are presently three key limitations to this protocol. The first limitation is that the protocol is technically challenging when using proteases due to the high propensity for cells to detach following the degradation of adhesive proteins and the extracellular matrix. Unfortunately, the use of poly-D-lysine coated coverslips does not overcome this issue. Some cell types may be amenable to a similar protocol using cells in suspension or following protease-mediated release from substrate. However, when U-2 OS cells were used following release into solution, their morphology was too poor to obtain reliable data.

The second limitation of this protocol is its current restriction to U-2 OS cells. While the protocol has not been tested in other cell types, it will likely transfer to other cell types with comparable adherence to U-2 OS cells.

The final limitation of this protocol is its reliance on fluorescently tagged proteins to determine the loss of protein targets from organellar assemblies. Visualizing a loss of signal from fluorescent proteins only suggests that the target protein has been degraded. Furthermore, the heterogeneity in permeabilization makes bulk determination of protein integrity uninformative. Thus, further support is required to demonstrate that protease treatment conclusively renders the target protein inactive. This support can take several forms. The strongest supporting evidence is when protein degradation results in an observable phenotype. For example, when cells are treated with proteinase K and the GFP-NPM1 signal is completely lost, the *snoRD3A* RNA disperses from its normal nucleolar localization, supporting that nucleolar proteins are required for the continued localization of this RNA (Figure 6). An alternative method to support observations from protease treatment experiments is pharmacological inhibition of the target protein. For instance, demonstrating that stress granules remain intact when G3BP1 dimerization is inhibited, rendering the protein inactive, supported the observation that G3BP1 is dispensable for stress granule RNA localization as determined by permeabilization and Proteinase K treatment. Similar approaches will ensure that the observations made by protease treating other assemblies are robust under various conditions.

## Troubleshooting

### Problem 1

Expression of fluorescently labeled marker proteins changes the behavior of the organelle of interest. Related to the “choosing a protein marker” section.

### Potential solution

Many membraneless organelles are sensitive to the concentration of their resident proteins and can form abnormally when proteins are overexpressed^11^^,^^16^. Several approaches can be used to mitigate this problem. When transfecting, reducing the expression of the exogenous protein by reducing the plasmid concentration or transfection duration can limit the effects of protein overexpression. Alternatively, using a low-efficiency promoter when designing vectors for the expression of fluorescently tagged proteins can also reduce protein abundance. Fluorescent proteins themselves can also affect some organelles. Changing the fluorescent protein can sometimes resolve issues with organelle formation. It is also recommended to either knock out the endogenous protein of interest, or knock it down and express the fluorescent version using an siRNA resistant vector. Finally, if all else fails, knocking in fluorescent proteins at the endogenous locus can minimize the effects of altering the protein of interest.

### Problem 2

Extracellular aggregates of fluorescent probes are visible with smiFISH probes. Related to the “annealing smiFISH probes” section.

### Potential solution

Occasionally, bright assemblies of smiFISH secondary probes are seen when using annealed smiFISH probe sets. These aggregates can typically be removed by spinning the annealed probes at high speed or reducing the amount of secondary probes used in annealing reactions to 0.8 μL.

### Problem 3

Poor degradation of RNA or protein. Related to Step 9 of the “permeabilization and enzyme treatment of cultured cells” section.

### Potential solution

When cells are permeabilized and enzyme-treated, getting good degradation of the target component can be challenging. A trial titration experiment is recommended to determine the efficiency of permeabilization and degradation. Treating U-2 OS cells with concentrations of PBSTween ranging from 0.2% to 1% for 10 min has correspondingly increased both the permeability and propensity for cells to detach in this protocol. Determining the optimum balance between these factors should be done on a case-by-case basis. Similarly, performing a time course of degradation can provide a better idea of the optimum fixation time. Ideally, these experiments will have a clear readout for satisfactory levels of degradation. For example, when stress granules are treated with RNases, G3BP1-GFP disperses from granules, and when they are treated with Proteinase K, the GFP signal is lost. Finally, titrating the degradative enzyme concentration can also improve degradation. Be aware that increasing protease concentrations also promote cell detachment, but higher RNase concentrations do not.

### Problem 4

There are very few cells remaining for imaging. Related to Step 11 of the “permeabilization and enzyme treatment of cultured cells” section.

### Potential solution

It cannot be overstated how tenuous cell adhesion to coverslips can be after protease treatment. If too many cells fall off during the protocol, try pipetting more slowly and against the wall of the wells during washes. It can also help to leave a moderate amount of solution above the coverslip to prevent the flow from adding solutions from directly shearing the sample. Additionally, be very delicate and minimize movement when handling coverslips for hybridization and slide preparation.

### Problem 5

Samples are drifting during imaging. Related to Step 47 of the “slide preparation and imaging” section.

### Potential solution

The samples can drift if too much solution is left between coverslips when making slides. Wick more solution away to remedy this issue.

### Problem 6

Low signal or fast photobleaching of FISH spots. Related to Step 47 of the “slide preparation and imaging” section.

### Potential solution

Single-molecule experiments such as RNA FISH can be very prone to photobleaching effects. If high-intensity illumination does not provide a satisfying signal to noise or if the signals disappear quickly during z-stack acquisition, signals may be bleaching. To remedy this problem, validate that the fluorophore and antifade combination work well for the sample. Staining for extremely bright signals, such as polyA RNAs during stress or nucleolar RNAs, can help differentiate between an issue with FISH protocols and photobleaching. If the FISH protocol is failing, no signal will be visible. If photobleaching is an issue, the signal should be present but rapidly disappear upon excitation. If a particular fluorophore bleaches too rapidly, use a different fluorophore or antifade solution (e.g., Prolong, n-propyl gallate solution.) Antifades are essential for preserving FISH signals, and excluding this component will almost certainly lead to photobleaching. Ambient light is not typically intense enough in comparison to laser excitation to cause noticeable photobleaching.^17^ Still, samples should generally be kept dark where possible.

### Problem 7

The best threshold in FISHquant either does not identify most FISH spots or misidentifies noise as spots. Related to Step 1, e, x of “analysis 1: Determination of RNA count and fraction in assemblies.”

### Potential solution

Robust signal-to-noise ratios are essential for effective automated spot detection in FISH-quant. If the intensity of FISH spots is less than ∼1.5-2 times the intensity of the background, FISH-quant will have difficulty accurately identifying spots. Maximizing the signal intensity achieved without bleaching FISH spots will make FISH-quant detections more effective.

### Problem 8

FISH-quant cannot identify individual RNA spots because they are overlapping in assemblies. Related to Step 1, e, x of “analysis 1: Determination of RNA count and fraction in assemblies.”

### Potential solution

The base implementation of FISH-quant is geared towards identifying single, discrete RNA signals. Previous work has developed methods to deconvolve these overlapping signals. The MATLAB script in analysis Step 2 will use a Gaussian mixture model to determine the approximate number and position of individual RNAs within larger foci.

## Resource availability

### Lead contact

For additional information, advice, and help troubleshooting, contact Dylan M. Parker (dylan.parker@colorado.edu).

### Technical contact

Questions about the technical specifics of performing the protocol should be directed to the technical contact, Dylan M. Parker (dylan.parker@colorado.edu).

### Materials availability

No new reagents were generated in this study.

### Data and code availability

• Due to file size constraints, data are available upon request.
• All scripts used to process data and nonstandard FISHquant MATLAB scripts have been deposited to Zenodo: https://doi.org/10.5281/zenodo.14662082.
• Any additional information required to reanalyze the data reported in this paper is available from the lead contact upon request.

## Acknowledgments

We thank the Parker lab, Paul Taylor, and Olke Uhlenbeck for the discussions. We also thank Florian Mueller and Erin Nishimura for their previous help in developing RNA FISH quantification pipelines. We thank the University of Colorado BioFrontiers Institute Cell Culture Facility, Advanced Light Microscopy Core Facility, Shared Instruments Pool Core Facility (RRID: SCR_018988, RRID: SCR_018302, and RRID: SCR_018986), and Paul Taylor for providing reagents, instruments, and advice. This work was funded by the Damon Runyon Cancer Research Foundation Postdoctoral Fellowship award, DRG 2471-22 (D.M.P.), and the Howard Hughes Medical Institute.

## Author contributions

Conceptualization, D.M.P. and R.P.; data curation, D.M.P.; formal analysis, D.M.P.; funding acquisition, D.M.P. and R.P.; writing, D.M.P. and R.P.

## Declaration of interests

The authors declare no competing interests.

## Supplemental information

Table S1. smFISH and smiFISH probes used in this study, related to Figures 3, 4, 5, and 6

## References

[1] Parker D.M., Tauber D., Parker R. (2025). G3BP1 promotes intermolecular RNA-RNA interactions during RNA condensation. Mol. Cell.

[2] Tsanov N., Samacoits A., Chouaib R., Traboulsi A.M., Gostan T., Weber C., Zimmer C., Zibara K., Walter T., Peter M. (2016). SmiFISH and FISH-quant - A flexible single RNA detection approach with super-resolution capability. Nucleic Acids Res..

[3] Femino A.M., Fay F.S., Fogarty K., Singer R.H. (1998). Visualization of single RNA transcripts in situ. Science.

[4] Figley M.D., Bieri G., Kolaitis R.-M., Taylor J.P., Gitler A.D. (2014). Profilin 1 Associates with Stress Granules and ALS-Linked Mutations Alter Stress Granule Dynamics. J. Neurosci..

[5] Wang W., Budhu A., Forgues M., Wang X.W. (2005). Temporal and spatial control of nucleophosmin by the Ran–Crm1 complex in centrosome duplication. Nat. Cell Biol..

[6] Rizzo M.A., Davidson M.W., Piston D.W. (2009). Fluorescent Protein Tracking and Detection: Fluorescent Protein Structure and Color Variants. Cold Spring Harb. Protoc..

[7] Tourrière H., Chebli K., Zekri L., Courselaud B., Blanchard J.M., Bertrand E., Tazi J. (2023). The RasGAP-associated endoribonuclease G3BP mediates stress granule assembly. J. Cell Biol..

[8] Kedersha N., Panas M.D., Achorn C.A., Lyons S., Tisdale S., Hickman T., Thomas M., Lieberman J., McInerney G.M., Ivanov P., Anderson P. (2016). G3BP–Caprin1–USP10 complexes mediate stress granule condensation and associate with 40S subunits. J. Cell Biol..

[9] Chen X., Bahrami A., Pappo A., Easton J., Dalton J., Hedlund E., Ellison D., Shurtleff S., Wu G., Wei L. (2014). Recurrent Somatic Structural Variations Contribute to Tumorigenesis in Pediatric Osteosarcoma. Cell Rep..

[10] Mittelman D., Wilson J.H. (2013). The fractured genome of HeLa cells. Genome Biol..

[11] Matsuki H., Takahashi M., Higuchi M., Makokha G.N., Oie M., Fujii M. (2013). Both G3BP1 and G3BP2 contribute to stress granule formation. Genes Cells.

[12] Mueller F., Senecal A., Tantale K., Marie-Nelly H., Ly N., Collin O., Basyuk E., Bertrand E., Darzacq X., Zimmer C. (2013). FISH-quant: automatic counting of transcripts in 3D FISH images. Nat. Methods.

[13] Majerciak V., Zheng Z.-M. (2025). Induction of translation-suppressive G3BP1+ stress granules and interferon-signaling cGAS condensates by transfected plasmid DNA. hLife.

[14] Imbert A., Ouyang W., Safieddine A., Coleno E., Zimmer C., Bertrand E., Walter T., Mueller F. (2021). FISH-quant v2: a scalable and modular analysis tool for smFISH image analysis. bioRxiv.

[15] Kowalczyk G.J., Cruz J.A., Guo Y., Zhang Q., Sauerwald N., Lee R.E.C. (2021). dNEMO: a tool for quantification of mRNA and punctate structures in time-lapse images of single cells. Bioinformatics.

[16] Banani S.F., Lee H.O., Hyman A.A., Rosen M.K. (2017). Biomolecular condensates: organizers of cellular biochemistry. Nat. Rev. Mol. Cell Biol..

[17] Samueli B., Kezerle Y., Dreiher J., Osipov V., Steckbeck R., Vaknine H., Baraban J.H. (2024). Shining Light on Photobleaching: An Artifact That Causes Unnecessary Excitation Among Pathologists. Arch. Pathol. Lab Med..

